# An underestimated factor for therapeutic decision-making in rare diseases: parents' (un)knowledge—the example of Duchenne muscular dystrophy caregivers and non-invasive ventilation

**DOI:** 10.1186/s13023-025-03762-9

**Published:** 2025-06-05

**Authors:** Eliza Wasilewska, Andrzej Wasilewski, Ewa Pilarska, Jolanta Wierzba, Sylwia Małgorzewicz, Marek Niedoszytko

**Affiliations:** 1 Department of Allergology, Medical University of Gdansk, 80-210, Gdansk, Poland; 2 Student Scientific Association of Medical Chemistry and Immunochemistry Wroclaw, Medical University Ul, M. Skłodowskiej-Curie 48/50 59-369, Wroclaw, Poland; 3 Department of Developmental Neurology, Department of Neurology, Medical University of Gdansk, Gdansk, Poland; 4 Department of Internal and Paediatric Nursing, Medical University of Gdansk, 80-210 Gdansk, Poland; 5 Department of Clinical Nutrition and Dietetics, Medical University of Gdansk, 80-210 Gdansk, Poland

**Keywords:** Duchenne muscular dystrophy, Neuromuscular diseases, Therapeutic decision-making, Parents’ knowledge, Pulmonary care, Non-invasive ventilation, Respiratory support, Pulmonary rehabilitation, Spirometry, Pulmonary function test

## Abstract

**Background:**

Duchenne muscular dystrophy (DMD) is a progressive genetic disease that leads to degeneration of muscles, including respiratory muscles, and requires early introduction of non-invasive ventilation (NIV). Parental knowledge and management strategies for pulmonary care are essential when respiratory function is compromised, particularly in conditions involving sleep apnea and the progression of chronic respiratory failure. The aim of this study was to assess parental knowledge of key aspects of pulmonary care in DMD and to identify knowledge gaps that may influence therapeutic decisions.

**Methods:**

A cross-sectional survey was conducted as part of the multidisciplinary care program for DMD at the Center for Rare Diseases in Gdańsk, accredited by the World Duchenne Organization. The questionnaire assessed (1) demographic and clinical details, (2) pulmonary healthcare practices, (3) understanding of pulmonary rehabilitation, (4) knowledge about NIV, and (5) sources of information on respiratory care.

**Results:**

The study included 111 parents (F/M 83/28, mean age 45.5 ± 6.75 years) of 111 children with DMD (all male, mean age 11.5 ± 5.45 years; 38% non-ambulatory). The majority of individuals (77.5%) regularly visited a pulmonary specialist with spirometry performed. Most parents reported satisfactory knowledge about respiratory issues in DMD but 77% of them reported insufficient knowledge about NIV (Chi^2^ = 53.4, df = 12, p = 0.00). Only 11% weren’t afraid to use NIV in the future, while 73% were afraid because of a lack of or inaccurate information. Physicians were the primary source of knowledge for pulmonary care, while the internet and peer experiences were rarely used.

**Conclusion:**

The majority of parents of children with DMD understand the basics of pulmonary problems. A significant information gap exists concerning advanced respiratory interventions such as non-invasive ventilation (NIV). This leads to anxiety among parents and impairs therapeutic decision-making, delaying appropriate treatment including respiratory support. There is a need for respiratory education programmes for parents and patients, especially as the estimated longer survival time for patients with DMD will make respiratory challenges even more significant.

**Graphical Abstract:**

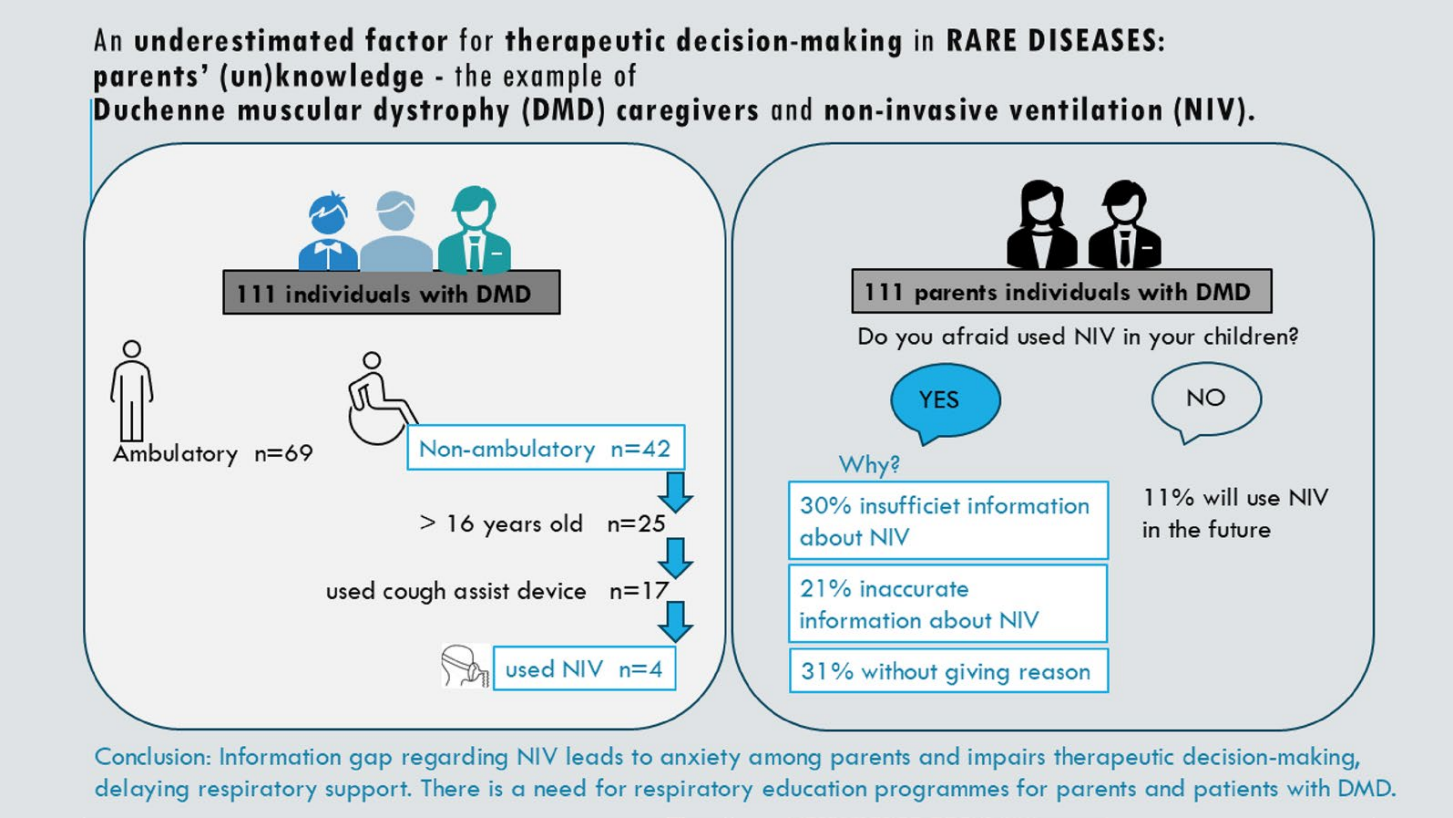

**Supplementary Information:**

The online version contains supplementary material available at 10.1186/s13023-025-03762-9.

## Introduction

Duchenne muscular dystrophy (DMD) is a severe, state X-linked neuromuscular disorder caused by mutations in the dystrophin gene. It is the most common and most severe form of inherited muscular dystrophy in children. It is characterized by progressive muscle degeneration leading to loss of motor function. In advanced stages, patients experience significant respiratory and cardiac complications, with respiratory failure being a major cause of morbidity and mortality in this population [1, 2].

Advances in healthcare such as corticosteroid therapy, multidisciplinary care, and new advanced therapies have extended the life expectancy of people with DMD, particularly into adulthood, resulting in new challenges, primarily related to respiratory function [3–5]. Progressive respiratory muscle weakness requires early and proactive respiratory management, including rehabilitation, airway clearance, and non-invasive ventilation (NIV), to reduce the risk of respiratory failure and its complications [6–9]. Timely initiation of NIV has been shown to improve quality of life, reduce hospitalizations, and prolong survival [10, 11].

However, the successful implementation of pulmonary care interventions depends not only on healthcare professionals but also on the role of caregivers, especially parents, who are often responsible for monitoring and managing the pulmonary care at home. Given the progressive nature of DMD and the complexity of respiratory interventions, caregiver knowledge and understanding of these therapies are important to achieving optimal patient outcomes [12]. Some research suggests that parents have insufficient knowledge and understanding of respiratory care, including the appropriate use of NIV [13–15].

The aim of this study was to assess the level of knowledge of a representative group of parents of children and adolescents with DMD regarding the important aspects of pulmonary care. In particular, the study aimed to determine parents' understanding of (1) pulmonary care management, (2) pulmonary rehabilitation strategies, (3) respiratory support—the use of non-invasive ventilation (NIV)—and (4) primary sources of information in these areas.

## Materials and methods

### Study design

The research project was a cross-sectional observational study conducted as part of the multidisciplinary care programme for patients with DMD at the Centre for Rare Diseases, University Clinical Centre (UCC), Medical University of Gdansk, Poland. The UCC is a member of the TREAT NMD Alliance Neuromuscular Network and is accredited by the World Duchenne Organisation.

Approval for the study was granted by the Ethics Committee No. NKBBN/260/2021 in accordance with the principles of the Declaration of Helsinki.

### Participants

The study included parents of males with DMD with previously confirmed diagnoses based on international standards including the presence of clinical symptoms, and genetic tests [1]. Participants were recruited during the 7th International Symposium “Possibilities of supporting the development of people with rare diseases—Duchenne Muscular Dystrophy and Other Muscular Dystrophies” organized by the Rare Disease Centre and the Parent Project Muscular Dystrophy Foundation, on 12–14 April 2023 in Sobieszewo, Poland. The conference concerned many aspects of the multidisciplinary care of DMD, including pulmonary care, dedicated to DMD patients with their families, caregivers, and medical professionals involved in DMD care [16].

Participants voluntarily in the survey, were informed of the purpose of the study, and were given full anonymity of their responses to minimize the impact of stress and anxiety associated with disclosing potential knowledge gaps. The survey was available online so participants could conveniently complete it from any mobile device or computer.

### Survey

All participants (DMD families; n = 153) were invited to complete the anonymous online survey (Google Forms https://forms.gle/RnCgZWhrVp1ma8oN7). Responses were opened on 12 April 2023 at 14:00 and closed on 30 April 2023 at 23:59. The questionnaires were completed in Polish via an online survey platform accessed via a link on the conference website.

The structured questionnaire consisted of closed and semi-open questions covering different areas of pulmonology healthcare and needs: (1) demographics and clinical status (2) pulmonary healthcare (3) pulmonary rehabilitation (4) respiratory support – NIV (5) sources of pulmonary knowledge.

1. Demographic data and clinical status were collected for parents (gender, age, and place of residence) and children (gender, age, weight, height, outpatient status, steroid therapy, type and number of respiratory infections, duration of infection, and number of hospitalizations), respectively.
2. In the pulmonary healthcare section, participants were asked about routine pulmonology consultations (unrelated to infection) and details of spirometry performed in the hospital and/or at home (e-spirometry monitoring—see [17, 18] for details).
3. Information on pulmonary rehabilitation included questions on the use and ownership of rehabilitation equipment.
4. The section on NIV support included questions on knowledge of respiratory problems in DMD, hypoventilation symptoms, and the use of mechanical ventilation. A five-point Likert scale (one—very poor, five—very good) was used to ensure the questions and responses were consistent. Parents who had concerns about the use of NIV were asked for their reasons.
5. Multiple-choice questions were used to collect information on parents' sources of knowledge about respiratory problems and support in patients with DMD.

### Statistical analysis

Statistical analyses of the data were performed using the Polish version of STATISTICA 13, PL. Descriptive analysis was performed to examine the differences between the distributions of categorical variables using the chi-square test. A P value < 0.05 was considered statistically significant. Data are presented as % and mean ± SD.

## Results

### Demographic data

#### Parents

Of the 153 families of children with DMD invited to the survey, a large group of 111 parents completed the questionnaire (Caucasian race, F/M 83/28, mean age 45.5 ± 6.75 SD), detailed data are presented in Table 1

**Table 1:** Sociodemographic, clinical data, and pulmonary care management

Parents n = 111 N (%) or Mean (SD)	
Mother/Father	83/28 (74.8%/25.2%)
Age	45.5 ± 6.75
Town of residence • < 10,000 • 10,000–100,000 • > 100,000	48 (43.3%) 34 (30.6%) 29 (26.2%)
DMD children n = 111	
Male	111 (100%)
Age	11.5 ± 5.45
Ambulatory/non-Ambulatory	69/42 (62.2%/37.8%)
Corticosteroids (current therapy)	100 (90.09%)
Lower Respiratory tract infections frequency (per year)	
• Never • 1 • 2 • > 2 Length • < 1 week • 1–2 weeks • > 2 weeks Hospitalization (per life) • Never • 1–2 • > 2	65 (58.6%) 26 (23.4%) 13 (11.7%) 7 (6.3%) 56 (50.4%) 48 (43.2%) 7 (6.3%) 97 (88.2%) 12 (10.8%) 2 (1.8%)
Routine pulmonology consultation (per year)	
• Never • < 1 • 1 • 2 • I don’t know	2 (1.8%) 6 (5.4%) 72 (64.8%) 16 (14.4%) 15 (13.5%)
Spirometry frequency (per year) • Never • < 1 • 1 • 2 place • During pulmonary consultation • Different, wherever on your own • Home spirometry monitoring	25 (22.5%) 9 (8.2%) 22 (19.8%) 55 (49.5%) 75 (67.6%) 11 (9.9%) 27 (24.3%)
Pulmonary rehabilitation device usage • Respiratory muscle training devices • Ambu bag recruitment of lung volume • Cough assist machine • Breathing exercises without devices o on your own o with a physiotherapist	39 (35.1%) 3 (2.7%) 17 (15.3%) 5 (4.5%) 21 (18.9%)
Non-invasive ventilation NIV usage Invasive mechanical ventilation	4 (3.6%) 0

#### Children

All children with DMD were male (Caucasian race, M 111, mean age 11.5 ± 5.45 SD); 37.8% were non-ambulatory; 90.1% were on steroid therapy (Table 1).

### Pulmonary care management

Details regarding pulmonary care management are presented in Table 1. Regarding routine pulmonology care, 89 patients (77.5%) reported attending regular consultations with a pulmonologist, typically one or two visits per year. However, 13 patients (11.7%) had no scheduled pulmonology visits, and two patients (1.8%) were not receiving any specialist care.

Spirometry was routinely performed in 77 patients (69.37%), with 55 undergoing the test twice a year and 22 annually. Conversely, 25 patients (22.5%) had never undergone spirometric evaluation. The mean age of patients who underwent spirometry testing (n = 86) was 12.97 ± 4.59 years, whereas the mean age of patients who had never undergone spirometry (n = 25) was 6.2 ± 3.15 years.

Spirometry was predominantly conducted at specialized pulmonary centers during consultations for 75 patients, while 11 patients performed spirometry independently.

Additionally, 27 patients (24.32%) reported using the remote home device "Aiocare" for monitoring respiratory function, while 62 patients (55.86%) indicated that they did not have access to the device but expressed interest in acquiring one for home use.

### Pulmonary rehabilitation

Pulmonary rehabilitation with the assistance of a physiotherapist was implemented in 21 patients (18.9%), while home-based rehabilitation was conducted by 29 parents (26.13%). Breathing exercise devices were utilized by 39 patients (35.1%). Breathing exercises without devices was performed in 5 patients (4.5%), and lung volume recruitment with an Ambu bag was employed in 3 patients (2.7%). A cough assistance device was used by 17 patients (15.3%) (see Table 1 and Table in supplementary for further details).

### Respiratory support – NIV

**Fig. 1:**
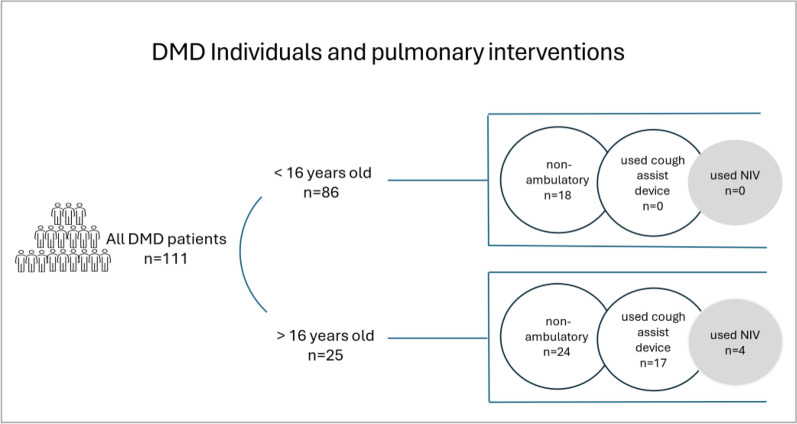
A discrepancy between the number of individuals who probably may require non-invasive ventilation (NIV) (individuals > 16 years old) and the number of individuals who used NIV (4 individuals)

Of all patients, 4 used NIV (nocturnal NIV), and there was a discrepancy between the number of patients using NIV and probably may require NIV (25 individuals) (Fig. 1).

**Fig. 2:**
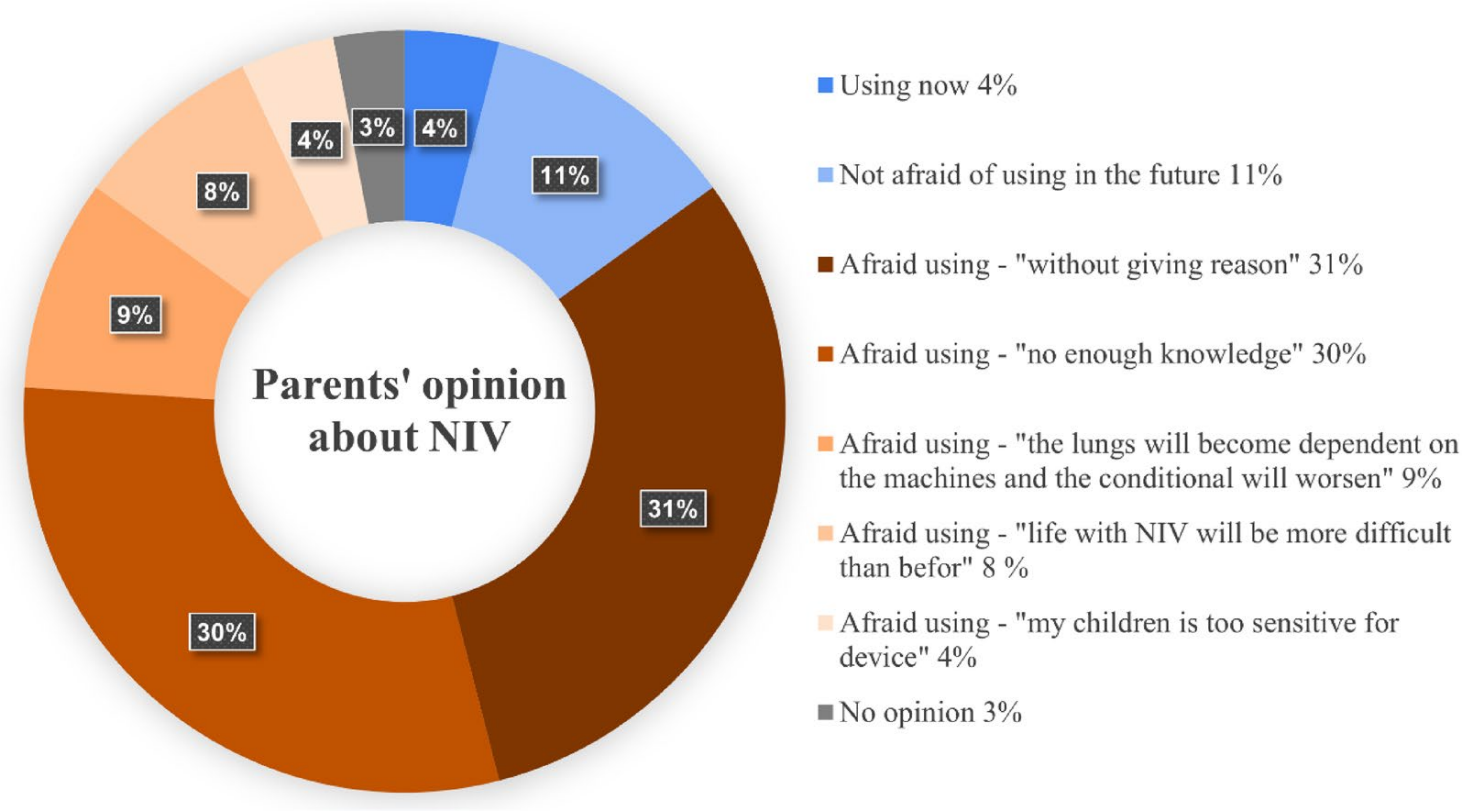
Caregivers’ opinions and anxiety associated with non-invasive ventilation (NIV)

Only 11% of parents were not afraid of using NIV in the future, while 31% of parents were fearful of using NIV without arguments, 30% of parents because of insufficient information about NIV; another justified the fear of using NIV with their own beliefs and arguments: "the lungs will become dependent on the machines and the condition will worsen" (9%), "live with NIV will be more difficult than before" (8%), "my children is too sensitive for device" (4%). See Fig. 2 for details.

### Parents’ knowledge

Table 2 shows detailed results of the level of knowledge of pulmonary issues held by parents of children with DMD. The parents presented adequate knowledge (very good and good) and insufficient knowledge (very poor, poor) regarding the natural history of the disease and the respiratory system in DMD (65% vs. 21.6%), symptoms of hypoventilation (54.9% vs. 31.5%), spirometry (45% vs. 27.9%), pulmonary rehabilitation (39.6% vs 50.4%), cough machine (29.7% vs 38.7%), NIV (21.6% vs 71.2%). The statistical analysis of results confirmed insufficient parents’ knowledge of NIV (Chi2 = 53.4, df 12;p = 0.00) and pulmonary rehabilitation (Chi2 = 130.1, df 16;p = 0.00).

**Table 2:** Results of parents' opinions on their level of knowledge about pulmonary issues. Data are presented as n = number of parents, % percentage of parents

Knowledge about	Very poor	Poor	No opinion	Good	Very good	Chi^2^; P value
Natural history of the respiratory system in DMD	6 (5.4%)	18 (16.2%)	14 (12.6%)	66 (59.5%)	7 (6.3%)	Chi^2^ = 116.4, df 16;p = 0.00
Symptoms of hypoventilation	12 (10.8%)	23(20.7%)	15(13.5%)	58 (52.2%)	3 (2.7%)	Chi^2^ = 197.3, df 16;p = 0.00
spirometry	6 (5.4%)	25 (22.5%)	30 (27.0%)	47 (42.3%)	3 (2.7%)	Chi^2^ = 126.3, df 16;p = 0.00
Pulmonary rehabilitation	8 (7.2%)	48 (43.2%)	11(9.1%)	32 (28.8%)	12 (10.8%)	Chi^2^ = 130.1, df 16;p = 0.00
Cough assist device	24 (21.6%)	19(17.1%)	35 (31.5%)	28 (25.2%)	5 (4.5%)	Chi^2^ = 125.0, df 16;p = 0.00
Non-invasive ventilation NIV	22 (19.8%)	57 (51.4%)	8 (7.2%)	24 (21.6%)	0	Chi^2^ = 53.4, df 12;p = 0.00

### Sources of Knowledge

Doctors were the primary source of information about the respiratory system (reported by 88 parents), symptoms of hypoventilation (64 parents), spirometry (72 parents), and cough assist devices (37 parents).

Physiotherapists were the primary source of knowledge about pulmonary rehabilitation (68 parents). The parents obtained the most information about the NIV from scientific conferences (70 parents).

Among parents of patients who could be on NIV (n = 25), the predominant sources of knowledge about respiratory support were physicians (52%) and parent conferences (32%).

**Fig. 3:**
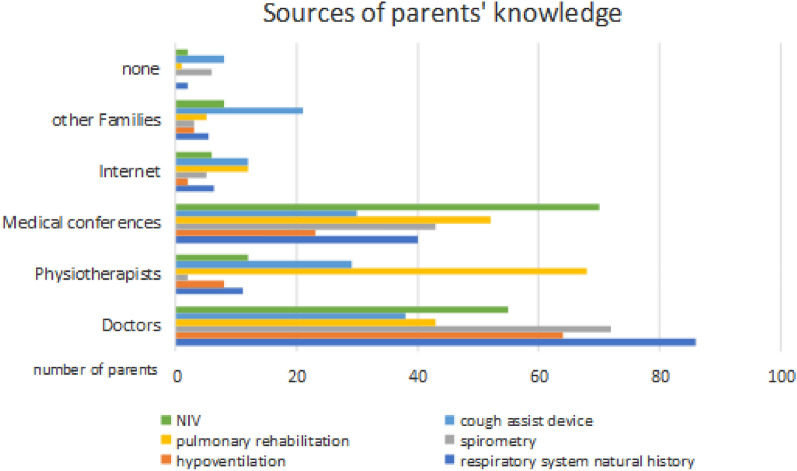
Sources of knowledge about respiratory issues in DMD based on parents' multiple-choice responses

The Internet and other families of children with DMD provided a secondary source of information, but among the analyzed topics parents most often looked for information on the Internet about cough assist devices and rehabilitation (12 parents respectively)—see Fig. 3.

## Discussion

Our study assessed the knowledge and understanding of pulmonary care among parents of children with DMD, focusing on identifying areas where further education is needed. To our knowledge, this is the first study involving such a large cohort of caregivers of individuals with DMD, representing over 7% of the estimated DMD population in Poland.

The most significant finding was that over 50% of parents rated their understanding of the respiratory system, hypoventilation symptoms, and spirometry satisfactory. Conversely, the majority of parents (approximately 80%) reported inadequate knowledge of NIV. Furthermore, a significant proportion (82%) expressed apprehension about using NIV for their children with DMD, which may impact the acceptance of this treatment.

In our study group, only four individuals used NIV, despite clinical data and age suggesting a likely higher prevalence of individuals requiring it (25 individuals). NIV is the preferred treatment for DMD to support weakened respiratory muscles and correct alveolar hypoventilation by maintaining adequate tidal volume and minute ventilation [19, 20]. It is recommended for hypoventilation, particularly in isolated nocturnal hypoventilation before the onset of daytime hypercapnia, obstructive sleep apnea, or when acute respiratory failure necessitates ventilatory support [21]. NIV has been shown to improve nighttime and daytime gas exchange, sleep quality, survival, and overall quality of life [22].

A review of the natural history reveals that the progression of hypoventilation symptoms accelerates following the onset of gait loss [2, 23]. NIV should be initiated once the essential relevant criteria, including gasometric, spirometric, and polysomnographic parameters, are met. [1, 6]. Additionally, the need for a cough assist device indirectly indicates a diminished cough reflex and potential hypoventilation, further supporting the necessity for NIV.

In our study cohort, 42 children were non-ambulatory; 25 were over 16 years old, and 17 had previously used a cough assist device at home. Theoretically, they required NIV support; however, only four had access to it in our group.

Czajkowska-Malinowska et al. reported that in Poland, relatively few children (regardless of diagnosis) use NIV (204 children) compared to adults (5,124 adults). The same study also highlighted a significant disparity in the use of NIV versus invasive ventilation (IV) across different age groups—only 30% of all mechanically ventilated children used NIV, compared to 73% in the adult group [24].

A notable finding in our study was parents' fear of using this treatment. While 11% of respondents indicated willingness to use NIV in the future, the majority of them expressed reservations. Among them, one-third did not provide specific reasons, while the remaining parents cited insufficient knowledge or personal beliefs as their main concerns. These included fears of poor treatment tolerance, reduced ability to continue daily activities, and even potential deterioration of lung function or dependence on the device.

A study involving a questionnaire completed by 10 DMD patients and their caregivers found that NIV improved daily functioning and breathing control. However, challenges included setting up the interface and managing battery life. Caregivers reported that while NIV provided reassurance and reduced anxiety, it also made caregiving more demanding due to the additional time required for equipment setup and monitoring [25].

Similarly, Patel et al. demonstrated that most patients generally prefer NIV, as it enhances mobility and independence [26]. Meanwhile, Pangalila et al. noted that tracheostomy is one of the potent factors contributing to increased caregiver burden in parents of children with DMD [27].

None of the surveyed patients underwent tracheostomy, but we do not have comprehensive data on the Polish population of DMD patients.

A study from Leeds, United Kingdom, reported a low prevalence of IV—only 3 out of 24 NIV-dependent patients with DMD underwent tracheostomy ventilation over a 17-year observation period [18].

It is important to note that, in general, adherence to NIV among DMD patients is poor, similar to trends seen in other chronic pediatric diseases [28]. Hurvitz et al. examined a group of pediatric and adult DMD patients aged 13 to 39 years requiring NIV and found significant variability in usage. While patients with lower lung function parameters used NIV for longer hours, overall adherence was inconsistent, and the reasons for non-compliance were unclear [29].

Our research revealed a significant discrepancy between the number of patients using NIV and those who likely require it. Additionally, we identified considerable parental apprehension regarding this treatment. This was an unexpected finding, given that most respondents considered pulmonary care for DMD patients as well-organized—more than 77% of patients were under regular pulmonological care, with systematic spirometry assessments.

This level of adherence to pulmonary monitoring is particularly noteworthy compared to a multicenter European study, where only 30% of patients had spirometry performed according to guidelines, especially among non-ambulatory patients [30]. The relatively high adherence in our study may partly result from selection bias, as parents who attend DMD conferences are often more engaged in specialized care. Additionally, the ongoing e-PULMoDMD home pulmonary telemonitoring project in Poland has likely increased awareness of spirometry’s importance among participating parents [17, 18]. Nonetheless, 10% of patients lacked an appropriately clear treatment plan, highlighting the need for more consistent and proactive respiratory care—especially since guidelines recommend pulmonology visits at least annually, increasing to biannual visits once patients lose independent mobility [1].

## Sources of pulmonary knowledge

Another key finding concerns the sources of information about pulmonary care. Most parents relied on healthcare professionals, with doctors being the primary source. However, a smaller proportion obtained information from medical conferences (27%), the Internet (5%), or other families with DMD children (5%). Surprisingly, the Internet—often a widely accessible resource for medical information—was underutilized in this context.

This is particularly unexpected given the widespread availability of the Internet in Poland and the abundance of DMD-related resources, such as **Parent Project Muscular Dystrophy (PPMD)**, which has over 1,600 followers [31]. Additionally, several websites and blogs, organized by the **World Duchenne Organization**, provide valuable support for families, though primarily in English [32]. Guidelines for parents of DMD children are also available in multiple languages, including Spanish, but not always in Polish [33–35].

Our findings highlight the need for more accessible and comprehensive educational materials on respiratory issues for parents, potentially through digital platforms or community-based interventions in their native language.

More than half of the parents of patients eligible for the NIV but not using it, cited doctors as the main source of knowledge. This is an interesting observation that would need to be investigated. Perhaps physicians should also have educational programs on what information and how to communicate with parents and patients at risk of respiratory failure and requiring respiratory support.

## Limitations

Despite the valuable insights gained, several limitations must be acknowledged. First, the study was conducted exclusively in Poland, which limits the possibility of generalizing the results to regions with different healthcare systems, cultural practices, and educational resources. Secondly, the reliance on self-reported data introduces the possibility of response bias, as parents may have over- or underestimated their knowledge. Lastly, participant recruitment occurred during a symposium and via an online survey, likely attracting more engaged caregivers, thus skewing the results toward a higher level of awareness.

Addressing these limitations in future studies could improve the robustness of findings and provide a more comprehensive understanding of DMD caregivers' educational needs. Previous research has primarily focused on the physical, mental, and emotional burdens faced by parents of children with DMD, such as stress, anxiety, and quality of life [36, 37]. Our study is the first to assess parents' knowledge of the respiratory system in DMD.

## Implications and conclusions

Despite the study’s limitations, our findings provide a foundation for developing educational programs to improve parents' and caregivers' knowledge of pulmonary issues. Enhancing awareness in this area could lead to better pulmonary care and reduction in respiratory complications among DMD patients.

- Most parents were aware of breathing problems in DMD.
- However, there was a significant knowledge gap regarding advanced respiratory interventions, such as **non-invasive ventilation (NIV)**. This gap contributed to parental anxiety and impaired therapeutic decision-making, delaying appropriate respiratory support.
- The Internet was an underutilized resource for pulmonary knowledge, indicating a need to make respiratory information more accessible, engaging, and widely promoted in the native language.
- There is an urgent need for **educational programs** tailored to these knowledge deficits, especially given the longer life expectancy of DMD patients resulting in increasing respiratory challenges they face.

These findings may also reflect cultural differences compared to other regions, further emphasizing the necessity of targeted educational interventions.

## Supplementary Information

Supplementary Material 1.

## Data Availability

All data generated or analyzed during this study are included in this published article in main text and supplementary materials.

## Abbreviations

DMD: Duchenne muscular dystrophy
NIV: Non-invasive ventilation
IV: Invasive ventilation
e-PULMoDMD: “E-monitoring of pulmonary function in patients with Duchenne Muscular Dystrophy” Project
PPMD: Parent Project Muscular Dystrophy
