# Supplementary material for: An underestimated factor for therapeutic decision-making in rare diseases: parents' (un)knowledge—the example of Duchenne muscular dystrophy caregivers and non-invasive ventilation
Source: Orphanet J Rare Dis. 2025 Jun 5;20:280. doi: 10.1186/s13023-025-03762-9 (PMC12143038; doi:10.1186/s13023-025-03762-9)
Supplement: Supplementary file 1 — Supplementary Material 1. [file 13023_2025_3762_MOESM1_ESM.docx]

*SUPPLEMENTARY MATERIAL*

Table S1. Basic information - raw data.

| No. | Content of the question | Answers |
| --- | --- | --- |
|  | Sex of the parent | Male- 28  Female- 83 |
| 1. . | Residency. | < 10 000 inhabitants – 48  10 000- 100 000 inhabitants – 34  >100 000 inhabitants- 29 |
|  | Parents’ age | mean±SD 45.5±6.75 range 27-56 |
|  | Children age | mean±SD 11.5 ± 5.45 range 3-24 |
|  | Sex of children | Male -111 |
|  | Glucocorticosteroid treatment | Yes- 100  No- 11 |
|  | How does the patient move? | By yourself - 69  Always a wheelchair - 42 |
|  | How many lower respiratory tract infections your child usually has in a year | Never – 65  1 time -26  2 times – 13  >2 times - 7 |
|  | Has the patient ever been hospitalised for a severe respiratory infection? | Yes, 1-2 times - 12  Yes, >2 times - 2  No - 97 |
|  | How long infections usually last in your child ? | Less than one week - 56  Between one and two weeks - 48  More than two weeks - 7 |

Table S2. Pulmonary healthcare section -raw data

| No. | Content of the question | Answers |
| --- | --- | --- |
| 1. . | Is your child under the systematic care of a DMD medical centre? | Yes – 99  No- 22 |
|  | Do you come for inspection systematically? | Yes- once a year - 72  Yes- twice a year - 16  Yes- less than once a year - 6  I do not have a visiting plan - 15  No - 2 |
|  | Does your child have a spirometry test done? | Never - 25  Sometimes (<1 time per year) - 9  Yes- once a year - 55  Yes- twice a year - 22 |
|  | If yes:  Where is spirometry performed: | During pulmonary consultation – 75  Different, wherever on your own – 11  Home spirometry monitoring - 27 |
|  | Do you know what FVC is? | Yes – 52  No - 59 |
|  | Do you know your child's FVC? | Yes – 23  No - 88 |
|  | Do you own an AioCare device? | Yes – 27  No, and does not plan to - 22  No, but I would like to have - 62 |

Table S3. Pulmonary rehabilitation - raw data.

| No. | Content of the question | Answers |
| --- | --- | --- |
|  | Does your child have pulmonary rehabilitation? | Yes – 48  No - 63 |
|  | If yes,  What kind of pulmonary rehabilitation device you use: | Breathing exercise devices – 39  Ambu bag recruitment of lung volume – 3  cough assist machine – 17  Manual recruitment of lung volume – 5  Only with physiotherapist - 21 |
|  | Do you know what a cough assistant is and what it does? | Yes – 38  No - 73 |
|  | Do you have a cough assistant at home ? | Yes – 17  No - 94 |
|  | Are you familiar with manual techniques to improve coughing? | Yes – 46  No - 65 |
|  | Do you use manual techniques to improve coughing? | Yes – 28  No - 83 |
|  | Do you know what mechanical assisted breathing is? | Yes – 77  No - 34 |

Table S4. Respiratory complications and support with NIV – raw data.

| No. | Content of the question | Answers |
| --- | --- | --- |
| 1. | DMD causes respiratory problems. | Yes-79  No-12  I don’t know -20 |
| 2. | If yes, What problems may occur in your child's respiratory system | recurrent respiratory infections -29  shortness of breath -52  shallow breathing - 57  coughing problems -49  dyspnea - 69 |
| 3. | Does your child use mechanical ventilatory support device? | Yes- non -invasive ventilatory (NIV) -4  Yes -invasive ventilatory (IV) -0  No- 107 |
| 4. | Are you aware of your child's breathing problems? | Yes – 59  No - 52 |
| 5. | Do you know when it is appropriate to think about non-invasive respiratory support in a child/adolescent? | Yes- 44  No- 67 |
| 6. | Do you agree with the following sentence: I know but am afraid to use non-invasive respiratory support? | Yes - 81  No – 25  I don’t know -5 |
| 7. | why are you concerned about the use of NIV in your child | without arguments 31  because of insufficient information about NIV – 27  because the lungs will become dependent on the machines and the condition will worsen -8  because live with NIV will be more difficult than before -7  because my children is too sensitive for device -3 |

Table S5. Parents’ sources of knowledge – multiple chois – raw data.

| No. | Content of the question | Answers |
| --- | --- | --- |
|  | Where did you get the information about possible respiratory problems ? | None - 2  Internet – 7  Physician – 52  Physioterapist -11  Conferences for parents – 45  Other families - 5 |
|  | How did you hear about non-invasive respiratory support ? | None - 26  Internet – 7  Physician – 55  Physioterapist - 12  Conferences for parents – 70  Other families - 7 |
|  | Where did you get the information about hypoventilation ? | None - 0  Internet – 27  Physician – 64  Physioterapist - 8  Conferences for parents – 23  Other families - 3 |
| 4. | Where did you get the information about spirometry? | None - 6  Internet – 5  Physician – 72  Physioterapist - 2  Conferences for parents – 43  Other families - 3 |
| 5. | Where did you get the information about pulmonary rehabilitation? | None -7  Internet – 27  Physician –43  Physioterapist - 68  Conferences for parents – 23  Other families - 3 |
| 6. | Where did you get the information about cough assistant device? | None - 8  Internet – 12  Physician – 38  Physioterapist - 68  Conferences for parents – 52  Other families - 21 |

Table S6. Clean data. Subjective assessment of the parent's knowledge expressed on a Likert scale.

| No. | Content of the question. | Likert score | | | | |
| --- | --- | --- | --- | --- | --- | --- |
|  |  | 1 | 2 | 3 | 4 | 5 |
| 1. | Assessment of general knowledge of the respiratory system. | 6 | 18 | 14 | 66 | 7 |
| 2. | Assessing knowledge of hypoventilation symptoms. | 12 | 23 | 15 | 58 | 3 |
| 3. | Assessing knowledge of spirometry. | 6 | 25 | 30 | 47 | 3 |
| 4. | Assessing knowledge of pulmonary rehabilitation. | 8 | 48 | 11 | 32 | 12 |
| 5. | Assessing knowledge of the cough device. | 23 | 18 | 36 | 29 | 5 |
| 6. | Assessing knowledge of non-invasive ventilation (NIV). | 22 | 56 | 8 | 25 | 0 |
